# Rethinking Biva in obesity: systematic vector displacement across the adiposity spectrum

**DOI:** 10.1038/s41430-026-01787-2

**Published:** 2026-07-28

**Authors:** Ricardo Rosero-Revelo, Mateo Tamayo, Daniel M. Rotroff, Kevin M. Pantalone, Bartolomé Burguera, Marcio L. Griebeler

**Affiliations:** 1 Endocrinology and Metabolism Department, Cleveland Clinic, Cleveland, OH USA; 2 Applied Medical Intelligence Unit, Fundación Santa Fe de Bogotá, Bogotá, DC Colombia; 3 Department of Quantitative Health Sciences, Lerner Research Institute, Cleveland Clinic, Cleveland, OH USA; 4 Center for Quantitative Metabolic Research, Cleveland Clinic, Cleveland, OH, USA

**Keywords:** Obesity, Body mass index

## Abstract

**Objective:**

To characterize Bioelectrical Vector Analysis (BIVA) vector patterns in adults with excess adiposity defined by fat mass index (FMI) and to examine the implications of obesity-related vector displacement for the clinical interpretation of BIVA.

**Methods:**

We conducted a retrospective cross-sectional analysis of adults attending a tertiary obesity clinic. Excess adiposity was defined using sex-specific FMI thresholds based on NHANES criteria. Resistance and reactance were normalized for height (R/H, Xc/H), and BIVA patterns were described overall and stratified by sex and ethnicity. Sex- and ethnicity-specific tolerance ellipses (50th, 75th, 95th percentiles) were derived from bivariate normal models and visually compared with Piccoli/NHANES reference ellipses for elevated adiposity. Correlation structure and ellipse geometry were evaluated within each sex–ethnicity stratum.

**Results:**

In 3072 adults with FMI-defined excess adiposity (723 men, 2349 women), impedance vectors showed a predominantly downward displacement characterized by lower Xc/H and lower phase angle, with variable R/H behavior across sex and ethnicity strata. This directional pattern was observed across the spectrum of FMI severity and remained stable across sex and major ethnic groups. Sex and ethnicity modulated mean vector position and ellipse dispersion but did not alter the overarching adiposity-related displacement. The correlation between R/H and Xc/H (r ≈ 0.63–0.75) was preserved in all strata, indicating intact underlying biophysical relationships.

**Conclusions:**

In adults with obesity, displacement of BIVA relative to classical reference ellipses primarily reflects the electrophysiological signature of excess adiposity rather than overt fluid overload or intrinsic cellular failure. These findings support the development of obesity-adjusted BIVA reference frameworks, further refined by sex and ethnicity, to improve the specificity and physiological interpretability of raw impedance measurements in obesity care.

**Figure:**
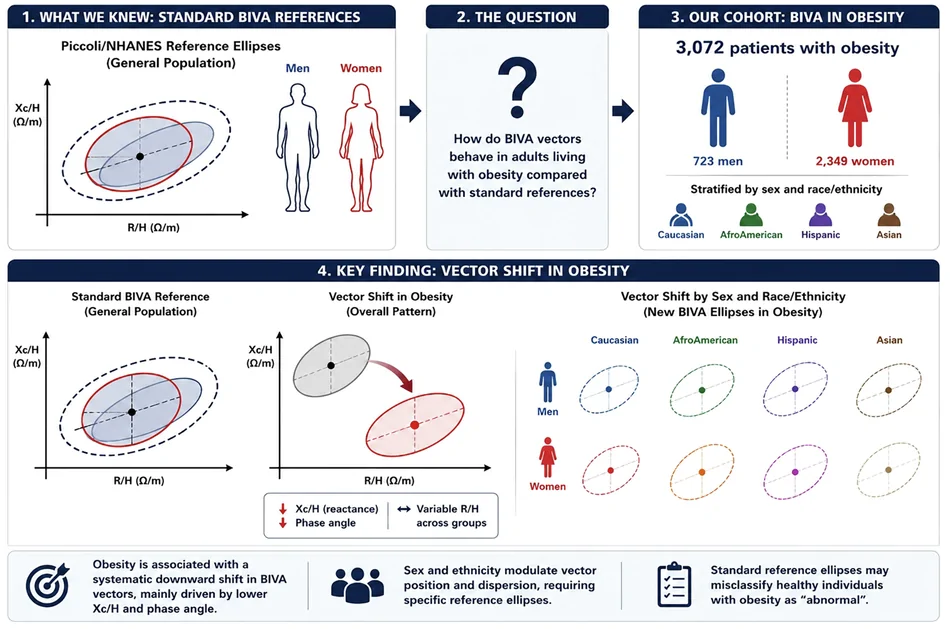

## Introduction

Obesity is a pathophysiologically heterogeneous disease in which individuals with similar body mass index (BMI) may differ substantially in fat mass (FM), lean tissue, and fluid distribution [1, 2]. Because BMI does not distinguish adipose from non-adipose mass and is insensitive to the electrical and functional properties of tissues, its capacity to capture the biological severity of obesity is limited [1, 3]. Fat mass index (FMI), which normalizes FM to height squared, provides a more physiologically grounded definition of excess adiposity and has been increasingly used to stratify obesity severity across sex and ethnic groups [4]. However, even FMI does not describe tissue integrity, hydration compartments, or conductivity, all of which are clinically relevant dimensions of chronic obesity.

Bioelectrical impedance vector analysis (BIVA) offers a complementary, equation-free approach to characterizing tissue physiology [5, 6]. By plotting resistance normalized to height (R/H) and reactance normalized to height (Xc/H), BIVA reflects whole-body conductivity, fluid distribution, and cell membrane capacitance without relying on regression-derived body composition estimates [7–10]. In Piccoli’s framework, resistance is predominantly determined by conductive pathways and hydration status, whereas reactance is influenced by membrane capacitance and the amount of metabolically active cell mass [7, 9]. These properties make BIVA sensitive to changes in fluid balance and cellular integrity and allow interpretation of vector displacement independently of body weight, BMI, or derived compartment estimates, enabling clinicians to infer, at a glance, whether a patient is broadly compatible with normohydration, dehydration or fluid overload and to obtain a qualitative impression of soft-tissue cellularity and integrity [11]. An alternative approach, termed specific BIVA, has been proposed to overcome the known limitations of classic BIVA in estimating relative FM. Unlike classic BIVA, which normalizes resistance and reactance by height, specific BIVA additionally accounts for body cross-sectional areas derived from segmental circumferences, thereby aligning the interpretation of impedance with Ohm’s law, according to which both conductor length and cross-sectional area influence impedance measurements. This approach has demonstrated improved accuracy for detecting FM percentage when compared with DXA [12]. However, specific BIVA requires anthropometric circumference data not routinely captured by most clinical impedance analyzers, which limits its applicability in standard care.

Reference tolerance ellipses derived from Piccoli provide a widely used framework for interpreting BIVA patterns in clinical practice [8]. However, these references were established primarily in normal-weight or overweight populations and may not accurately represent the vector geometry of individuals with chronic, FMI-defined excess adiposity. In obesity, higher adipose mass, alterations in extracellular-to-intracellular water ratios, and reduced cellular density could attenuate Xc/H and reduce phase angle, while also modifying ellipse orientation and dispersion. Of note, studies using specific BIVA—which normalizes impedance not only by height but also by cross-sectional body areas—have reported longer vectors in individuals with obesity and sarcopenic obesity, a pattern attributable to correction for the geometric effect of larger body transverse areas on resistance [13, 14]. It remains unclear to what extent such shifts reflect true pathological changes—such as fluid overload or impaired cellular integrity—versus the expected electrical signature of longstanding excess adiposity. Furthermore, sex and ethnicity influence fat distribution, skeletal muscle mass, hydration compartments, and membrane properties, suggesting that BIVA patterns in obesity may exhibit systematic, demographically driven variation [7, 15–17].

To address these gaps, we conducted a BIVA-based analysis in a large cohort of adults with excess adiposity defined by sex-specific FMI thresholds. The primary objective was to characterize BIVA vector patterns (R/H and Xc/H) in this FMI-defined excess-adiposity population and to compare sex- and ethnicity-specific tolerance ellipses with established Piccoli/NHANES reference ellipses [7]. Beyond this primary aim, we sought to derive sex- and ethnicity-specific 50th, 75th and 95th percentile tolerance ellipses for R/H and Xc/H in adults with excess adiposity, to quantify directional differences in mean impedance components between these obesity-specific ellipses and Piccoli/NHANES reference ellipses for elevated adiposity and to characterize how the correlation structure and orientation of vectors vary across sex–ethnicity strata. Finally, we aimed to interpret these obesity-specific BIVA patterns in terms of their implications for the clinical reading of impedance vectors in patients with chronic excess adiposity.

## Materials and methods

### Study design and population

This was a retrospective cross-sectional analysis of a clinical body-composition database derived from routine bioelectrical impedance assessments performed with the seca mBCA 552 analyzer at a single tertiary care center. The source dataset included all available impedance measurements with linked demographic and anthropometric information. For individuals with repeated assessments, only the earliest measurement was retained to avoid intra-individual dependence. Body composition parameters included height, weight, FMI, and raw impedance measurements (resistance and reactance). Fat mass, fat-free mass (FFM), total body water (TBW), and extracellular water (ECW) were estimated by the manufacturer-embedded algorithms of the seca mBCA 552 analyzer, which apply population-specific, multi-frequency bioelectrical impedance equations validated against reference methods. Phase angle, reported by the device as arctan(Xc/R) × 180/π at 50 kHz, was used as provided. The seca mBCA 552 is a multi-frequency, phase-sensitive bioelectrical impedance analyzer that operates at frequencies between 1 and 1000 kHz using an 8-electrode configuration (4 electrodes on each hand and foot). Measurements are performed in a standing position with the patient barefoot on the platform and grasping the hand electrodes. The device has been validated against reference methods, including DXA and deuterium dilution [6, 18]. Reproducibility data from the manufacturer report a coefficient of variation <1% for resistance and reactance measurements under standardized conditions.

Excess adiposity was defined using sex-specific FMI thresholds (FMI ≥ 6 kg/m² in men and ≥9 kg/m² in women), consistent with NHANES-based reference ranges for elevated adiposity [4]. The present analysis was restricted to patients with excess adiposity by FMI, complete impedance data, and a recorded ethnicity; ethnicity-stratified analyses focused on individuals classified as Caucasian, AfroAmerican, or Hispanic. These groupings were based on self-reported categories recorded in the clinical database and are used here as analytical variables reflecting population-level differences in body composition and tissue electrical properties, not as biological determinants, consistent with current recommendations on the reporting of race and ethnicity in medical research [19]. After applying these criteria, the final BIVA excess-adiposity cohort comprised 3,072 individuals. For BIVA, height was converted to meters and impedance was normalized by height to derive resistance-to-height (R/H) and reactance-to-height (Xc/H), expressed in ohms per meter (Ω/m), which were used in all subsequent analyses.

### Statistical analysis

All analyses were performed separately by sex and, when sample sizes allowed, further stratified by ethnicity (Caucasian, AfroAmerican, and Hispanic). Age and FMI were summarized as mean ± standard deviation and as median with interquartile range (P25–P75). For the BIVA variables (R/H and Xc/H), we computed sex- and ethnicity-specific means, standard deviations, and Pearson correlation coefficients, and additionally reported global summary statistics for the overall high-adiposity cohort to contextualize vector position. Using these parameters, sex- and ethnicity-specific covariance matrices for R/H and Xc/H were estimated, and tolerance ellipses corresponding to the 50th, 75th, and 95th percentiles were derived from a bivariate normal model using χ² quantiles. For each sex–ethnicity stratum, these clinical high-adiposity ellipses were plotted alongside Piccoli/NHANES reference ellipses for individuals with elevated adiposity, allowing direct visual comparison of mean vector location and ellipse orientation between the clinical cohort and reference populations. Mahalanobis distances from each individual vector to the corresponding sex–ethnicity mean were computed to assign observations to probability regions consistent with the 50%, 75%, and 95% ellipses, providing a qualitative assessment of concordance and displacement relative to reference BIVA patterns. Differences between cohort means and Piccoli/NHANES reference means in R/H and Xc/H were summarized numerically (absolute and directional deltas) and categorized descriptively as “similar,” “higher,” or “lower” based on pre-specified magnitude thresholds. All data management, BIVA computations, and graphical outputs were performed in Python version 3.14 (Python Software Foundation) using the pandas (data handling), NumPy (numerical operations), SciPy (matrix and distance calculations), and matplotlib (graphics) libraries.

## Results

In this FMI-defined excess adiposity cohort of 3072 adults (723 men and 2349 women), mean BMI was 35.4 ± 7.1 kg/m² and mean FMI 16.5 ± 5.9 kg/m², with an average fat mass of 45.3 ± 16.3 kg, fat-free mass of 52.8 ± 11.0 kg, total body water of 39.0 ± 7.9 L and an ECW/TBW ratio of 47.3 ± 3.3%. Mean phase angle was relatively low at 5.1 ± 0.7°, consistent with impaired cellular integrity despite preserved total mass. According to FMI categories, 24.2% of participants were classified as “excess fat”, 28.1% as obesity class I, 22.1% as class II and 25.6% as class III, indicating broad dispersion across the adiposity spectrum. Although BMI did not differ significantly by sex, men were taller and had substantially higher FFM, SMM and TBW, whereas women had higher FM and FMI (all *p* < 0.001). FMI classes also showed sex-related shifts, with men more frequently classified as class III and women more often in class I–II, underscoring that, even within a uniformly high-BMI cohort, the partitioning of mass into fat and lean compartments is strongly sex-dependent (Table 1).

**Table 1:** Body-composition and impedance characteristics of the FMI-defined excess-adiposity cohort by sex.

	Male (*n* = 723)	Female (*n* = 2349)	*p*-value
	Mean ± SD or *n* (%)	Mean ± SD or *n* (%)	
Height (cm)	176.2 ± 7.5	163.2 ± 6.7	<0.001
Resistance, R (Ω)	494.5 ± 67.8	601.0 ± 75.8	<0.001
Reactance, Xc (Ω)	47.6 ± 9.4	52.5 ± 9.2	<0.001
Resistance/height, R/H (Ω/m)	281.1 ± 40.8	368.9 ± 48.9	<0.001
Reactance/height, Xc/H (Ω/m)	27.0 ± 5.5	32.3 ± 5.8	<0.001
Age	53.6 ± 14.4	51.5 ± 13.9	<0.001
Body mass index (kg/m²)	35.5 ± 7.2	35.4 ± 7.0	0.783
Fat mass (kg)	42.5 ± 17.5	46.2 ± 15.8	<0.001
Fat mass index (kg/m²)	13.7 ± 5.6	17.3 ± 5.8	<0.001
Fat-free mass (kg)	67.8 ± 9.3	48.2 ± 6.5	<0.001
Skeletal muscle mass (kg)	32.0 ± 5.2	21.3 ± 3.7	<0.001
Total body water (L)	49.6 ± 6.9	35.8 ± 4.7	<0.001
Extracellular water (L)	22.1 ± 3.3	17.2 ± 2.5	<0.001
ECW/TBW (%)	44.5 ± 2.4	48.2 ± 3.0	<0.001
Phase angle (degrees)	5.5 ± 0.8	5.0 ± 0.7	<0.001
FMI obesity class, n (%)			
Excess fat	141 (19.5%)	603 (25.7%)	
Obesity class I	185 (25.6%)	679 (28.9%)	
Obesity class II	157 (21.7%)	522 (22.2%)	
Obesity class III	240 (33.2%)	545 (23.2%)	
Ethnicity, n (%)			
African American	90 (12.4%)	459 (19.5%)	
Asian	15 (2.1%)	24 (1.0%)	
Caucasian	594 (82.2%)	1,783 (75.9%)	
Hispanic	24 (3.3%)	83 (3.5%)	

Ethnicity further modulated these body-composition profiles. Among men, Caucasian and AfroAmerican groups had similar height (~176.6 cm), whereas Hispanic men were shorter (168.9 ± 10.1 cm) and exhibited higher mean R/H and Xc/H values, indicating a more displaced impedance vector. Kruskal–Wallis tests showed that, in men, FFM, SMM, TBW, ECW and ECW/TBW differed significantly across Caucasian, AfroAmerican and Hispanic groups, while BMI, FM and FMI did not, suggesting that ethnic gradients are driven more by lean and fluid compartments than by BMI-defined adiposity alone. In women, ethnicity-related contrasts were even more pronounced: Caucasian, AfroAmerican and Hispanic women had broadly similar height ranges but significantly different BMI, FM, FMI, FFM, SMM, TBW, ECW, ECW/TBW and PhA (all *p* < 0.001, except Xc/H). Within women, Caucasians showed a balanced distribution across FMI classes, AfroAmerican women had a lower proportion in “excess fat” but a higher proportion in class III, and Hispanic women concentrated more frequently in class II, highlighting ancestry-specific patterns in the severity spectrum of FMI-defined obesity (Table 2).

**Table 2:** Body composition and BIVA characteristics by ethnicity and sex (FMI-defined excess adiposity).

	Caucasian (*n* = 594)	AfroAmerican (*n* = 90)	Hispanic (*n* = 24)	Asian (*n* = 15)	*p*-value
	Mean ± SD	Mean ± SD	Mean ± SD	Mean ± SD	
Males					
Height (cm)	176.6 ± 7.3	176.6 ± 6.8	168.9 ± 10.1	171.0 ± 7.2	<0.001
Resistance (Ω)	494.9 ± 63.4	475.8 ± 72.4	495.5 ± 93.1	590.1 ± 86.8	0.021
Reactance (Ω)	47.3 ± 9.0	47.1 ± 10.4	48.5 ± 12.2	59.7 ± 9.0	0.548
R/H (Ω/m)	280.7 ± 37.4	269.6 ± 41.3	295.3 ± 65.8	346.3 ± 55.3	0.016
Xc/H (Ω/m)	26.8 ± 5.2	26.7 ± 6.0	28.8 ± 7.6	35.0 ± 5.6	0.249
BMI (kg/m²)	35.3 ± 6.8	37.0 ± 9.2	35.2 ± 8.3	30.8 ± 7.6	0.504
Fat mass (kg)	42.7 ± 16.8	43.8 ± 20.9	38.7 ± 19.7	34.1 ± 18.1	0.234
Fat mass index (kg/m²)	13.7 ± 5.3	14.1 ± 7.1	13.5 ± 6.6	11.5 ± 5.8	0.671
Fat-free mass (kg)	67.7 ± 8.8	71.5 ± 9.5	62.4 ± 12.2	56.7 ± 10.1	<0.001
Skeletal muscle mass (kg)	32.0 ± 5.0	34.3 ± 5.2	28.4 ± 7.0	27.0 ± 5.8	<0.001
Total body water (L)	49.7 ± 6.5	51.8 ± 6.9	45.0 ± 9.0	41.2 ± 7.6	0.001
Extracellular water (L)	22.1 ± 3.1	23.3 ± 3.5	20.6 ± 4.0	17.5 ± 3.4	<0.001
ECW/TBW (%)	44.4 ± 2.3	44.9 ± 2.6	45.8 ± 3.4	42.6 ± 3.4	0.022
Phase angle (°)	5.46 ± 0.82	5.65 ± 0.87	5.59 ± 1.04	5.81 ± 0.73	0.220
r (R/H, Xc/H)	0.635	0.716	0.728	0.664	
	**Caucasian (***n* = **1783)**	**AfroAmerican (***n* = **459)**	**Hispanic (***n* = **83)**		***p*-value**
	Mean ± SD	Mean ± SD	Mean ± SD	Asian (*n* = 24)	
Females				Mean ± SD	
Height (cm)	163.4 ± 6.6	163.3 ± 6.8	158.7 ± 6.9	160.3 ± 4.3	<0.001
Resistance (Ω)	604.5 ± 74.9	583.5 ± 74.8	602.6 ± 79.7	666.0 ± 78.1	<0.001
Reactance (Ω)	52.3 ± 9.0	52.7 ± 9.6	54.5 ± 9.6	60.2 ± 8.2	0.052
R/H (Ω/m)	370.6 ± 48.5	357.8 ± 46.6	380.5 ± 53.6	416.0 ± 51.3	<0.001
Xc/H (Ω/m)	32.1 ± 5.7	32.3 ± 6.0	34.4 ± 6.1	37.6 ± 5.3	0.001
BMI (kg/m²)	34.9 ± 7.0	37.6 ± 7.0	35.5 ± 6.5	31.5 ± 4.9	<0.001
Fat mass (kg)	45.5 ± 15.7	49.7 ± 16.4	44.4 ± 13.8	37.8 ± 9.8	<0.001
Fat mass index (kg/m²)	17.0 ± 5.7	18.6 ± 5.9	17.6 ± 5.2	14.7 ± 3.7	<0.001
Fat-free mass (kg)	47.7 ± 6.3	50.6 ± 6.4	45.3 ± 6.7	43.1 ± 4.5	<0.001
Skeletal muscle mass (kg)	21.0 ± 3.6	22.9 ± 3.8	19.5 ± 3.6	19.5 ± 2.5	<0.001
Total body water (L)	35.6 ± 4.6	37.1 ± 4.7	33.3 ± 4.8	32.0 ± 3.4	<0.001
Extracellular water (L)	17.1 ± 2.5	18.1 ± 2.4	16.5 ± 2.5	14.7 ± 1.8	<0.001
ECW/TBW (%)	48.0 ± 2.9	49.0 ± 3.0	49.5 ± 2.9	45.8 ± 2.0	<0.001
Phase angle (°)	4.95 ± 0.64	5.16 ± 0.71	5.16 ± 0.61	5.17 ± 0.43	<0.001
r (R/H, Xc/H)	0.677	0.679	0.750	0.829	

**Fig. 1:**
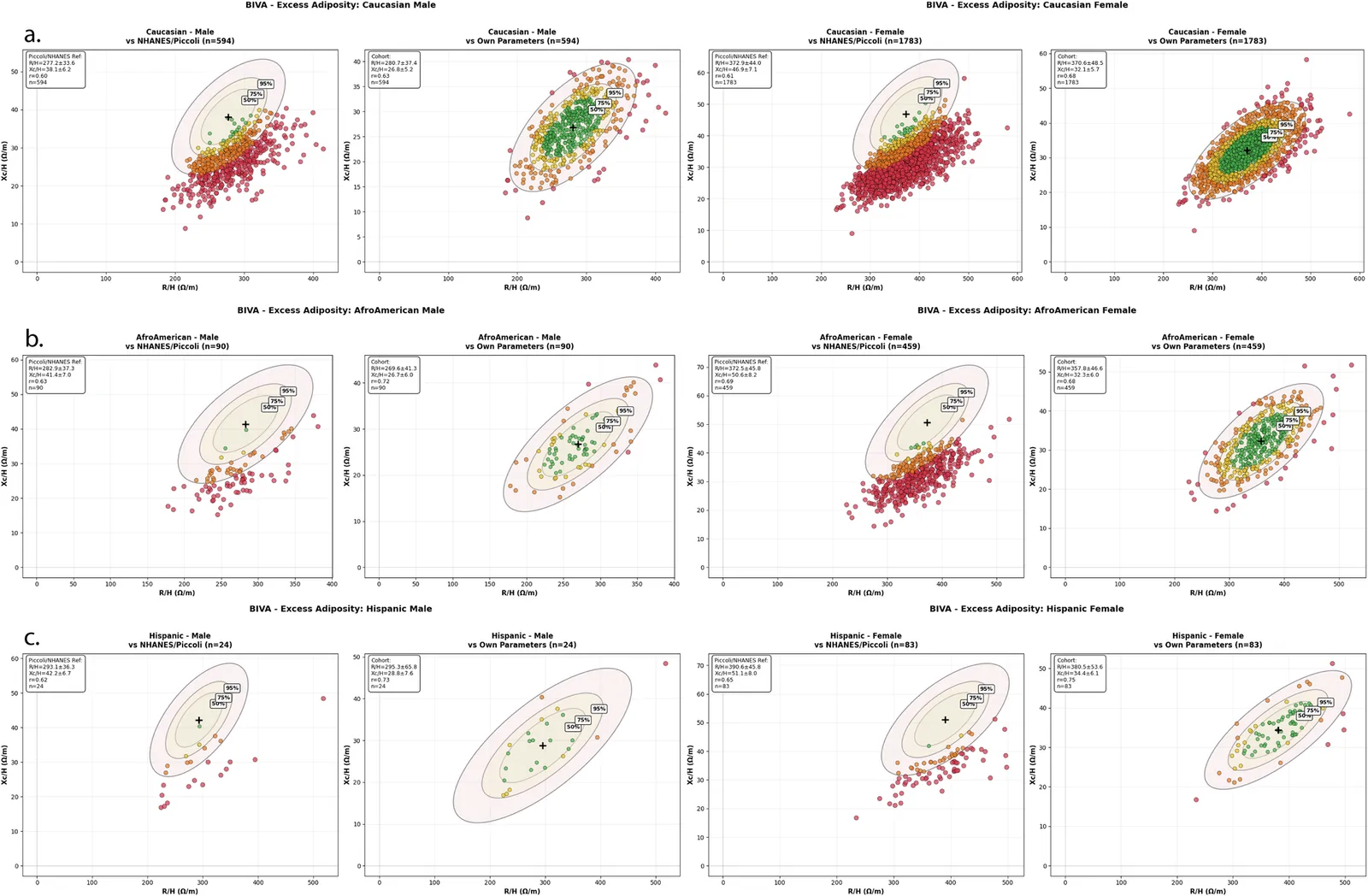
Sex- and ethnicity-specific BIVA patterns in adults with FMI-defined excess adiposity. Caucasian (**a**), AfroAmerican (**b**) and Hispanic (**c**) men and women. For each sex–ethnicity group, the left subpanel overlays individual vectors from the excess-adiposity cohort (red dots) on the corresponding Piccoli/NHANES 50th, 75th and 95th tolerance ellipses (solid lines), whereas the right subpanel displays cohort-derived ellipses based on the present study (dashed lines). Crosses (+) indicate Piccoli/NHANES reference centers, and circles (○) indicate cohort centers. All vectors are expressed as resistance/height (R/H) and reactance/height (Xc/H) in Ω/m.

**Fig. 2:**
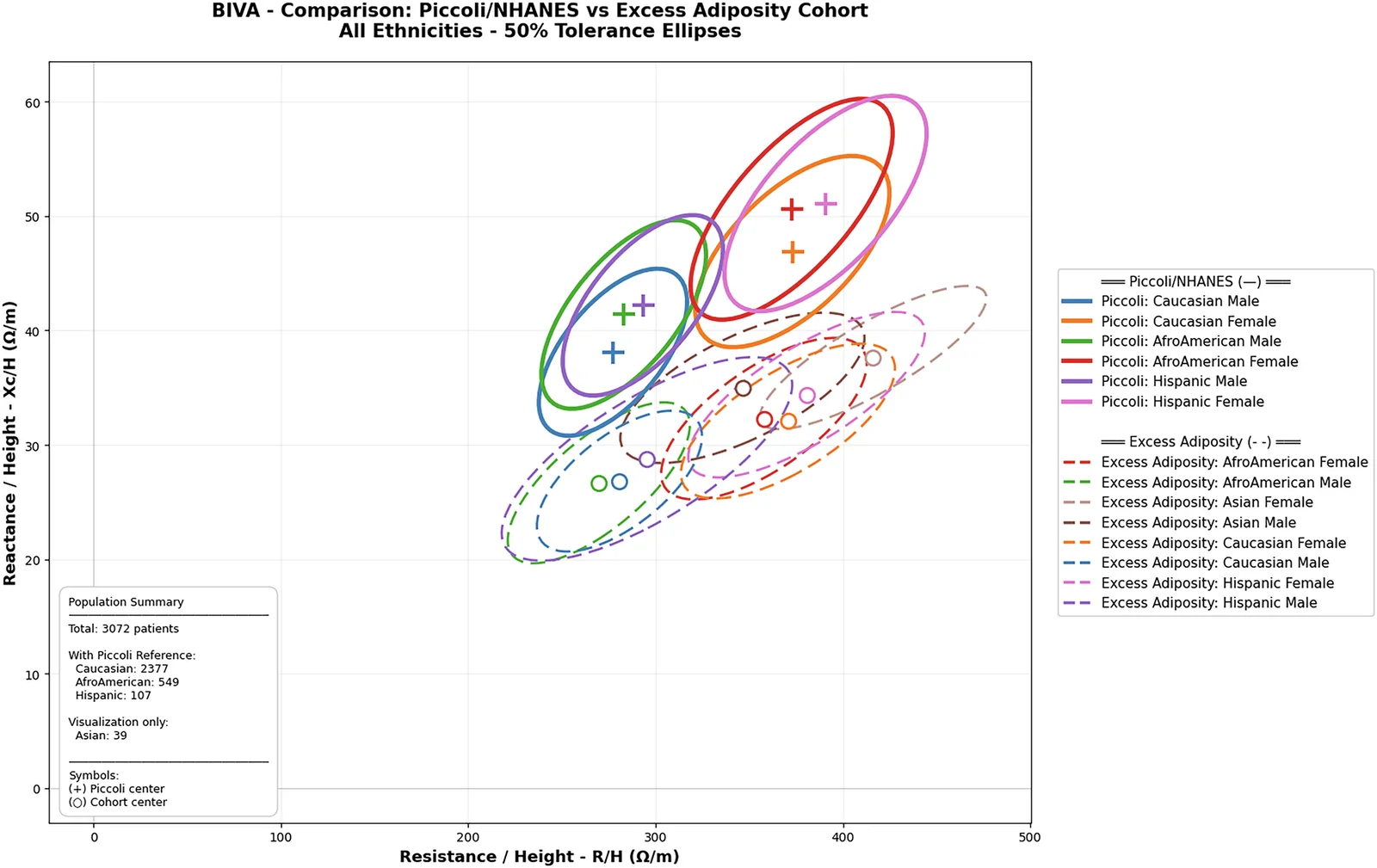
Comparison of 50% BIVA tolerance ellipses from Piccoli/NHANES references and the FMI-defined excess-adiposity cohort across sex and ethnicity. Solid ellipses represent Piccoli/NHANES 50% tolerance regions for Caucasian, AfroAmerican and Hispanic men and women; dashed ellipses depict the corresponding 50% regions derived from the excess-adiposity cohort. Crosses (+) denote Piccoli/NHANES mean vectors and circles (○) denote cohort means. Asian men and women are shown for visualization only (no available Piccoli/NHANES reference), illustrating their relative position within the impedance plane.

Bioelectrical impedance vector analysis (BIVA) confirmed coherent, sex- and ethnicity-specific shifts of the mean vector (R/H, Xc/H) and corresponding tolerance ellipses. Our cohort showed a predominantly downward displacement of the mean vector, reflected by lower Xc/H values and consistent with lower phase angle, while shifts in R/H were directionally variable across sex and ethnicity. The correlation between R/H and Xc/H remained strong within each sex–ethnicity stratum (r ≈ 0.63–0.75), preserving the characteristic elliptical structure of BIVA (Figs. 1 and 2).

## Discussion

This study was designed to address a recurring observation in routine obesity care: clinically stable patients with FMI-defined excess adiposity were systematically plotted outside classical BIVA tolerance ellipses, despite the absence of overt nutritional or fluid imbalance [13, 14, 20]. Using a large, well-characterized cohort and sex- and ethnicity-specific ellipses anchored in FMI rather than BMI, we show that this apparently “abnormal” positioning is not random noise but a reproducible, obesity-specific vector pattern. Across the full range of FMI severity, and in both sexes and all ethnic groups with sufficient sample size, excess adiposity was more consistently associated with a downward vector displacement, reflected by lower Xc/H values, whereas shifts in R/H were less uniform across strata. Taken together, these findings support the hypothesis that chronic adiposity itself is likely the primary driver of vector displacement, and that the apparent misclassification arises from applying reference frameworks that do not incorporate the geometry of obesity.

From a biophysical standpoint, the obesity-related displacement of BIVA vectors observed in this cohort is highly plausible and is better interpreted as a predominantly downward shift characterized by lower Xc/H and lower phase angle, with variable R/H behavior across sex and ethnicity strata [21, 22]. Adipose tissue has lower electrical conductivity than lean tissue, so as FM expands, the fraction of poorly conductive tissue per unit body volume increases, and the number of effective conductive pathways decreases, even in clinically stable individuals. In our cohort, however, this did not translate into a uniform increase in R/H, supporting the interpretation that R/H behavior is modified by sex, ethnicity, body geometry, and hydration distribution rather than by adiposity alone [22]. At the same time, chronic obesity is characterized by a relative dilution of metabolically active cell mass within a larger body volume and by extracellular water expansion, along with subtle changes in membrane composition [23–28], which may attenuate, rather than augment, membrane capacitance. As a result, lower Xc/H appears to be the more consistent feature, producing the predominantly downward vector displacement observed in our cohort, whereas changes in R/H were more variable across sex and ethnicity. This lower Xc/H pattern is compatible with relative extracellular water expansion, which may reduce the capacitive contribution of cell membranes to whole-body reactance [29]. Consistently, the lower phase angle observed in the cohort further supports this interpretation, since PhA reflects the proportional relationship between reactance and resistance and is inversely related to the extracellular-to-intracellular water ratio, while also being influenced by membrane capacitance and tissue cellularity [30]. Taken together, these mechanisms are consistent with a “bioelectrical phenotype” of excess adiposity rather than an intrinsic failure of the impedance signal.

Stratified analyses by sex and ethnicity further refined this picture, showing that the obesity-related vector displacement sits on top of structured demographic differences in body composition. Men displayed higher fat-free mass, skeletal muscle mass and total body water than women, while women had higher FM and FMI [31–33], and these contrasts translated into sex-specific BIVA geometry with distinct mean vector locations and ellipse dispersion. Across ethnic groups, differences in lean mass distribution, extracellular-to-intracellular water ratios and tissue architecture [7, 16, 17, 34–37] produced additional shifts in ellipse centers and orientations—particularly evident between Caucasian, AfroAmerican, Hispanic and Asian subgroups. However, these demographic effects acted as secondary modifiers of a shared pattern: in all sex–ethnicity strata with adequate sample size, vectors showed a predominantly downward displacement characterized by lower Xc/H and variable R/H behavior, while the correlation between resistance and reactance remained strong. This preservation of correlation structure suggests that the core biophysical relationships captured by BIVA remain intact in obesity; what changes is the baseline position of the “normal” vector cloud for each demographic context.

These observations have direct implications for clinical interpretation. In current practice, BIVA is frequently used to infer hydration status, tissue quality and cellular integrity and vectors located below classical ellipses are often read as signals of fluid imbalance, inflammation or impaired cell integrity [8, 9, 38–42]. Our data suggest that, in individuals with chronic excess adiposity, a predominantly downward vector displacement with variable R/H behavior is more consistent with the expected electrical signature of high FMI than with overt pathological decompensation, although the cross-sectional design of this study does not allow definitive exclusion of subclinical fluid or cellular alterations. Adjusting BIVA reference ellipses to account for obesity, and further refining them by sex and ethnicity, would allow clinicians to distinguish expected adiposity-related vector displacement from clinically meaningful deviations. In practical terms, when interpreting BIVA in patients with obesity, clinicians should first recognize that vectors may lie outside classical reference ellipses as a consequence of chronic excess adiposity itself. Therefore, an isolated downward displacement should not be interpreted as fluid overload, inflammation, or impaired cellular integrity without considering FMI, sex, ethnicity, device protocol, and the patient’s clinical context. Future studies should evaluate the diagnostic performance of these obesity-adapted frameworks in terms of sensitivity, specificity and predictive value for different clinically relevant alterations in hydration, cellular integrity and body composition to determine how BIVA can be most effectively integrated into routine obesity care.

Finally, these findings should be interpreted considering the study’s strengths and limitations. Strengths include the large, clinically derived cohort, standardized impedance measurements obtained under routine care conditions, and the use of FMI-based criteria rather than BMI alone to define excess adiposity. The consistency of vector patterns across sex and major ethnic groups reinforces the robustness of an obesity-specific electrical phenotype. However, the single-center, cross-sectional design precludes causal inference and limits generalizability to other care settings and devices, and sample sizes for some ethnic strata were modest, which may reduce precision in estimating ellipse geometry. Impedance measurements in this study were obtained with a seca mBCA 552 analyzer in a standing position, whereas the Piccoli/NHANES reference data were collected with a Valhalla Scientific Model 1990B in a supine position. Measurement posture may affect raw impedance values and phase angle, which may contribute to the absolute magnitude of the downward displacement observed, but does not invalidate the directional pattern [21]. Moreover, differences among BIA devices in technology, electrode configuration, and measurement position can result in significant inconsistencies in impedance values, and direct comparison of absolute values across devices should be interpreted with caution [43–45]. We did not disentangle the effects of specific comorbidities (e.g., heart failure, chronic kidney disease, inflammatory disorders) or pharmacologic therapies on vector position, nor did we examine longitudinal changes or clinical outcomes.

Although specific BIVA—which normalizes bioelectrical variables by cross-sectional body areas derived from segmental circumferences—has demonstrated improved accuracy for detecting relative FM, the anthropometric circumferences required for its computation were not available in this clinical dataset. Recent evidence further supports the need for population-specific bioelectrical vector references, showing that vectors from individuals with obesity may fall outside the 95% tolerance ellipse of the general adult population when assessed using specific BIVA [46]. Moreover, DXA-based validation of impedance-derived FM introduces its own systematic errors in individuals with obesity [47–49]. Because the present study does not aim to estimate FM from impedance but rather to evaluate the adequacy of classical BIVA reference ellipses for individuals with chronic excess adiposity, the classic BIVA framework was retained. Future work should validate obesity-adjusted, sex- and ethnicity-informed BIVA references in independent cohorts—ideally with device-specific ellipses— explore how comorbidities and obesity phenotypes (central vs peripheral, sarcopenic obesity) further reshape vector geometry, and test whether these adapted frameworks improve risk stratification, monitoring of nutritional and fluid status, and response to interventions in people living with obesity.

## Conclusion

Adults with FMI-defined excess adiposity exhibit consistent and physiologically meaningful shifts in bioelectrical impedance vectors that differ substantially from classical reference patterns. These vector displacements arise from the altered hydration, conductivity, and cellular properties characteristic of chronic obesity and should not be interpreted using ellipses derived from normal-weight or mixed-weight populations. Sex and ethnicity further modulate vector geometry, underscoring the need for population-specific and disease-specific reference frameworks. BIVA remains a valuable physiological tool in obesity, but its interpretation requires recalibration to avoid misattributing adiposity-related electrical patterns to pathological fluid shifts or impaired cellularity. Establishing obesity-specific BIVA ellipses is therefore essential to support accurate clinical assessment and future integration of raw impedance metrics into obesity phenotyping, and future studies should formally evaluate the diagnostic performance (sensitivity, specificity and predictive value) of these adapted frameworks for detecting true decompensation, fluid overload and sarcopenic or inflammatory remodeling in people living with obesity.

## Data Availability

The dataset analyzed in this study contains clinical information that cannot be made publicly available due to institutional policies on patient data protection. De-identified data may be made available from the corresponding author upon reasonable request and following appropriate regulatory and institutional approvals.
